# MSCHLMDA: Multi-Similarity Based Combinative Hypergraph Learning for Predicting MiRNA-Disease Association

**DOI:** 10.3389/fgene.2020.00354

**Published:** 2020-04-15

**Authors:** Qingwen Wu, Yutian Wang, Zhen Gao, Jiancheng Ni, Chunhou Zheng

**Affiliations:** ^1^School of Software, Qufu Normal University, Qufu, China; ^2^School of Computer Science and Technology, Anhui University, Hefei, China

**Keywords:** microRNA, disease, miRNA-disease association, K-nearest neighbor, K-means, combinative hypergraph learning

## Abstract

Accumulating biological and clinical evidence has confirmed the important associations between microRNAs (miRNAs) and a variety of human diseases. Predicting disease-related miRNAs is beneficial for understanding the molecular mechanisms of pathological conditions at the miRNA level, and facilitating the finding of new biomarkers for prevention, diagnosis and treatment of complex human diseases. However, the challenge for researchers is to establish methods that can effectively combine different datasets and make reliable predictions. In this work, we propose the method of Multi-Similarity based Combinative Hypergraph Learning for Predicting MiRNA-disease Association (MSCHLMDA). To establish this method, complex features were extracted by two measures for each miRNA-disease pair. Then, K-nearest neighbor (KNN) and K-means algorithm were used to construct two different hypergraphs. Finally, results from combinative hypergraph learning were used for predicting miRNA-disease association. In order to evaluate the prediction performance of our method, leave-one-out cross validation and 5-fold cross validation was implemented, showing that our method had significantly improved prediction performance compared to previously used methods. Moreover, three case studies on different human complex diseases were performed, which further demonstrated the predictive performance of MSCHLMDA. It is anticipated that MSCHLMDA would become an excellent complement to the biomedical research field in the future.

## Introduction

MicroRNAs(miRNAs) are a class of small endogenous non-coding RNAs that mainly regulate gene expression at the post-transcriptional level, whose length is equivalent to 20–25 nucleotides (Bartel, 2009; Ribeiro et al., 2014). The first miRNA was discovered in the early 1990's. However, miRNAs were not recognized as a distinct class of biological regulators until the early 2000's. Recently, accumulating studies have indicated that more than one-third of genes are regulated by miRNAs (Taguchi, 2012), and that miRNAs participate in various biological processes, such as cell proliferation, tissue development, apoptosis, differentiation and signal transduction (Mattick and Makunin, 2006; Esteller, 2011; Mattick and Rinn, 2015). The deregulation of miRNAs appears to be associated with various diseases, ranging from common diseases to cancers (Sayed and Abdellatif, 2011; Farazi et al., 2013). For example, based on deep sequencing information and cluster analysis, several miRNAs, including miR-7, miR-95, miR-124, miR-128, and miR-132 were found to be significantly down-regulated in glioblastoma (Skalsky and Cullen, 2011). In addition, Dkk-3 and SMAD4 were identified as potential target genes of miR-183, and the expression of miR-183, miR-146a, and miR-767-5P were significantly higher in prostate cancer tissues (Ueno et al., 2013).

Therefore, predicting potential miRNA-disease associations could not only improve our knowledge of the underlying disease mechanisms at the miRNA level, but also facilitate the finding of novel disease biomarkers for early detection and drug discovery in the contexts of disease prevention, diagnosis, treatment and prognosis. However, compared with the rapidly increasing number of newly discovered miRNAs, only a few miRNA-disease associations have been confirmed. Experimental confirmation of the new disease-related miRNAs is extremely expensive and time-consuming, whose failure rate is also high. Currently, a great quantity of biological data about miRNAs has been generated, and more and more studies have focused on the computational algorithms which can select the most promising miRNAs for further analysis. By decreasing the number of experiments, more effective experimental procedures could be conducted to uncover potential disease-related miRNAs on a large scale.

Mainstream computational methods are roughly grouped into two categories. The first category is based on network analysis (Chen et al., 2012, 2016; Zeng et al., 2016, 2018; Li et al., 2017; Liu et al., 2017; Xiao et al., 2017; Zhong et al., 2017). Jiang et al. (2018) designed the significance SIG of disease pairs or miRNA pairs and then developed a novel miRNA-disease association prediction (ICFMDA) method, which was used to improve the collaborative filtering approach. The collaborative filtering algorithm was further improved by incorporating similarity matrices to enable the prediction of a new miRNA and a particular disease without known associations. Chen et al. (2018) proposed a Two-tier Random Walk method in which they designed a Laplacian score of graphs for the prediction of disease-related miRNAs (GSTRW). This method can predict the correlation of all diseases with miRNAs simultaneously without negative samples. By performing a depth-first search algorithm on the heterogeneous network to infer disease-related miRNAs, You et al. (2017) presented a model called PBMDA, which could be employed in new diseases or miRNAs, greatly improving practicability and reliability. Chen et al. (2018c) designed a Network Distance Analysis method for miRNA-disease Association prediction (NDAMDA), which used the direct network distance and average network distances between two miRNAs or diseases. However, this model might cause a bias toward miRNAs with more known related diseases and might not be applicable to the diseases where associated miRNAs tend to be randomly distributed in the network. Zhao Q. et al. (2018) developed a miRNA-disease association prediction method based on the Spy and super clustering strategy (SSCMDA). They used a Spy strategy to recognize trustworthy negative samples from the uncertain miRNA-disease pairs which could improve prediction accuracy. However, this method used the Regularized Least Square as the baseline classifier and it was difficult to attain the optimal combining parameters to merge all the developed strategies. Zhao H. C. et al. (2018) proposed a method to predict miRNA-disease associations based on a distance correlation set (DCSMDA). The high point of this approach lay in the construction of a miRNA-lncRNA-disease network that could be applied to predict potential lncRNA-disease associations. Nevertheless, this approach cannot be applied to unknown diseases or miRNAs that are not present in the miRNA-disease or lncRNA-miRNA databases. Later, Zhao et al. (2019) developed a method based on a shortest path algorithm for discovering potential miRNA-disease associations. This method improved the sparseness of known associations and did not require negative samples to predict potential miRNA-disease association simultaneously.

Methods that belong to the second category are adopted machine learning algorithms used to predict miRNA–disease associations (Jiang et al., 2010; Xu et al., 2011; Chen et al., 2015). Chen and Yan (2014) designed a semi-supervised method called RLSMDA. This method could identify disease-related miRNAs without known miRNAs. However, the parameter optimization for RLSMDA was challenging. Chen et al. (2018a) proposed a new machine learning method for miRNA-disease association prediction. They used a stacked auto-encoder to extract deep features and a greedy unsupervised algorithm for a pre-training model. At last, the support vector machine (SVM) was utilized to uncover potential associations. However, the optimization of complex parameters was complicated in this model. Furthermore, Chen et al. (2018b) designed a prediction method named EGBMMDA, which adopted an Extreme Gradient Boosting Machine to predict potential associations. This approach was the first decision tree learning-based method and one of the very few models that achieved a global LOOCV AUC >0.9 at that time. Recently, Xuan et al. (2019) developed a dual convolutional neural network-based method for predicting potential disease-miRNA association (CNNMDA), which was a computational model based on deep learning and used the original and global representation of an miRNA-disease pair to predict disease-related miRNAs. However, this method has many parameters and involves a large number of calculations.

Although the methods mentioned above have made great contributions to the discovery of miRNA-disease associations, there are still some limitations in many aspects. In addition, the limited number of known miRNA-disease associations results in a sparse matrix. Thus, in order to improve the accuracy of the prediction model, we propose a novel prediction method based on a hypergraph and refer to it as MSCHLMDA. The edge of a hypergraph can own more than two vertices, endowing hypergraphs with high flexibility for depicting high-order relationships. Benefitted by this desirable property, hypergraph models have been successfully applied to dozens of computer vision as well as machine learning and pattern recognition areas. The performance of hypergraph learning highly depends on the generated hypergraph structure. A good hypergraph structure can represent the data correlation better. In this study, for all the miRNA-disease pairs, two different measures (graph theoretical and statistical) were utilized to formulate the potential informative features, and a combinative hypergraph learning model was designed to predict their unknown associations. Experiments with cross validations and case studies fully demonstrated that the performance of our method in predicting the potential disease-miRNA associations has a significant advantage compared to previous methods.

## Materials and Methods

### Method Overview

Our model mainly consists of two steps: (I) data collection and preprocessing, (II) association prediction. First, the feature vector *X* of all miRNA-disease pairs was constructed; Second, the combinative hypergraph model was designed to learn projection matrices, which were used to map the unknown miRNA-disease pair features to the association scores matrix *S*.

### Data Collection

The raw data used by our method were three matrices: miRNA-disease association matrix *A*, miRNA similarity matrix *SM* and disease similarity matrix *SD*. Matrix *A* was obtained from the HMDDv2.0 (Li et al., 2014), which contains 5,430 known associations between 495 (*nm*) miRNAs and 383 (*nd*) diseases. Concretely, if miRNA *m*(*i*) is verified to be associated with disease *d* (*j*), the value of *A* (*m* [i], *d* [*j*]) is equal to 1, and 0 otherwise. Our goal is to predict the link between miRNAs and diseases in matrix *A. SM* was directly downloaded from http://www.cuilab.cn/files/images/cuilab/misim.zip. It included similarity scores for all 495 miRNAs, for which the scores were calculated according to the Wang et al. (2010) method. The larger the *SM* (m [*i*], m [*j*]) is, the closer their associations will be.

SD contains the similarity scores of different diseases. Based on the disease classification system in the Mesh database, we can use a directed acyclic graph (DAG) to describe the similarity between different diseases. There were two methods to calculate the contribution values of disease *d* (*t*) to the semantic value of disease *d* (*i*) as follows:

(1) $$\begin{array}{l} {D1}_{d\left(i\right)}\left(d\;\left(t\;\right)\right)=-\log\left(\frac{the\;number\;of\;DAGs\;including\;d\left(t\right)\;}{the\;number\;of\;diseases}\right) \end{array}$$

(2) $$\begin{array}{l} DV1\left(d\;\left(i\;\right)\right)\;=\sum_{d\left(t\right)\in T\left(d\left(i\right)\right)\;}{D1}_{d\left(i\right)}\left(d\left(t\right)\right) \end{array}$$

and

(3) $$\begin{array}{l} D2_{d(i)}(t)\\ =\{\begin{array}{l} \;1\text{ if }d(t)=d(i)\;\\ \max\left\{\Delta^{*}D2_{d\left(i\right)}\left(d(t^{\prime })\right)|d(t^{\prime })\in \text{child of }d\left(t\right)\right\}\text{if }d\left(t\right)\neq d\left(i\right) \end{array} \end{array}$$

(4) $$\begin{array}{l} DV2\left(d\;\left(i\;\right)\right)\;=\sum_{d\left(t\right)\in T\left(d\left(i\right)\right)\;}{D2}_{d\left(i\right)}\left(d\left(t\right)\right) \end{array}$$

where Δ represents the semantic contribution factor. It will reduce the contribution of disease *d* (*t*) if *d* (*t*) is different from *d* (*i*).

The disease similarity score was calculated based on the measurement of common subgraphs between disease DAGs. So, the similarity between disease *d* (*i*) and *d* (*j*) could be defined as below:

(5) $$\begin{array}{l} SD1\left(d\;\left(i\;\right),d\;\left(\;j\;\right)\right)\;=\frac{\sum_{t\in T\left(d\left(i\right)\right)\cap T\left(d\left(j\right)\right)}\left({D1}_{d\left(i\right)}\left(t\right)+{D1}_{d\left(j\right)}\left(t\right)\right)}{DV1\left(d\left(i\right)\right)+DV1\left(d\left(j\right)\right)} \end{array}$$

and

(6) $$\begin{array}{l} SD2\left(d\;\left(i\;\right),d\;\left(\;j\;\right)\right)\;=\frac{\sum_{t\in T\left(d\left(i\right)\right)\cap T\left(d\left(j\right)\right)}\left({D2}_{d\left(i\right)}\left(t\right)+{D2}_{d\left(j\right)}\left(t\right)\right)}{DV2\left(d\left(i\right)\right)+DV2\left(d\left(j\right)\;\right)} \end{array}$$

Therefore, by integrated *SD*1 and *SD*2, we could reconstruct a new similarity matrix *SD* = $\frac{SD1+SD2}{2}$.

### Data Preprocessing

Generally, the similarity of miRNAs, as well as the similarity of diseases, is used to predict the association between miRNAs and diseases directly. However, some unknown interactions might affect the prediction results. To address this limitation, the WKNNP preprocessing method (Xiao et al., 2017) was used to estimate previously unknown but possible interactions between miRNAs and diseases through their known neighbors in the matrix *A*. If the value of *A* (i,j) is 0, the role of WKNNP is to update it to a value in the range of 0 to 1. Then the complete matrix A is used to generate Gaussian interaction profile kernel (Gipk) similarity (Laarhoven et al., 2011). For miRNAs, a vector *KS* (*m*[*i*]), i.e., the *i*-th row of matrix *A*, was utilized as the interaction profiles of miRNA *m* (*i*) for denoting the association between *m* (*i*) itself and each disease. Thus, the Gipk similarity *GIM* (*m* [*i*], *m* [*j*]) of miRNA *m* (*i*) and miRNA *m* (*j*) was defined as:

(7) $$\begin{array}{l} GIM\left(m\left(i\right),\;m\left(j\right)\right)\;=\;exp\left(-\gamma_{m}\;∥\;KS\left(m\left(i\right)\right)\;-\;KS\left(m\left(j\right)\right){∥}^{2}\right) \end{array}$$

Where ||·*||*^2^ represented *l*_2_ norm, γ_*m*_ was a parameter used to control the kernel bandwidth, which was set as

(8) $$\begin{array}{l} \gamma_{m}=\frac{1}{\frac{1}{nm}\sum_{i=1}^{nm}∥KS\left(m\left(i\right)\right){∥}^{2}} \end{array}$$

By integrating *SM* and *GIM*, a more comprehensive miRNA multi-similarity matrix *MMS* could be obtained as

(9) $$\begin{array}{l} MMS\left(m\left(i\right),\;m\left(j\right)\right)\\ =\;\{\begin{array}{l} GIM\left(m\left(i\right),\;m\left(j\right)\right)\;if\;SM\left(m\left(i\right),\;m\left(j\right)\right)=\;0\;\\ \frac{SM\left(m\left(i\right),\;m\left(j\right)\right)+GIM\left(m\left(i\right),\;m\left(j\right)\right)}{2}\;otherwise\; \end{array} \end{array}$$

Similarly, we also calculated the Gipk similarity *GID* for diseases by the follow formulas

(10) $$\begin{array}{l} GID\left(d\left(i\right),\;d\left(j\right)\right)\;=\exp\left(-\gamma_{d}\;∥\;KS\left(d\left(i\right)\right)\;-\;KS\;\left(d\left(j\right)\right){∥}^{2}\right) \end{array}$$

(11) $$\begin{array}{l} \gamma_{d}=\frac{1}{\frac{1}{nd}\sum_{i=1}^{nd}∥KS\left(d\left(i\right)\right){∥}^{2}} \end{array}$$

where *KS* (*d* (*i*)) and *KS* (*d* (*j*)) denoted the *ith* column and the *jth* column of *A*. At last, the disease multi-similarity matrix *DMS* was obtained by

(12) $$\begin{array}{l} DMS\left(d\left(i\right),\;d\left(j\right)\right)\\ =\{\begin{array}{l} GID\left(d\left(i\right),\;d\left(j\right)\right)\;if\;SD\left(d\left(i\right),\;d\left(j\right)\right)=\;0\;\\ \frac{GID\left(d\left(i\right),\;d\left(j\right)\right)+SD\left(d\left(i\right),\;d\left(j\right)\right)}{2}\;otherwise\; \end{array} \end{array}$$

In the above process, all known miRNA-disease associations in matrix *A* would be used to calculate the GipK similarity. Therefore, before the cross validation, the corresponding value of a known miRNA-disease association in matrix *A* should be set to 0, if it was a test sample.

### Feature Construction

Based on the description of the literature (He et al., 2017), there were three types of features to be constructed. Type 1 features summarized *A, MMS* and *DMS* from a statistical perspective. For miRNA *m* (*i*)/disease *d* (*j*), we calculated

- *num*. *ass*: the number of known association in *A* (*i*,:)/*A* (:, *j*).
- *me*. *sim*: for *m*(*i*), the mean of *MMS* (*i*,:); for *d* (*j*), the mean of *DMS* (*j*,:).
- *dis*. *sim*: calculate the distribution of similarity scores for *m* (*i*)/*d* (*j*). Here, the similarity scores were divided into 5 parts.

Type 2 features described *MMS*/*DMS* using graph theories. Graphs for miRNAs and diseases from *MMS* and *DMS* were built, respectively. The nodes were representing miRNAs or diseases; if two nodes' similarity scores were greater than the mean value of all entities in *MMS*/*DMS*, they would be linked by an edge. For each node, we defined the following features

- *num*. *nb*: number of neighbors.
- *k*. *sim*: the similarity values of the k-nearest neighbors of the node (in our study *k* equal 20).
- *bt, cl*: betweenness, closeness of the node.

Type 3 features focused on matrix *A*. We defined the following features for each miRNA-disease pair based on statistics and graph theories.

- *m*. *d*. *nb*: the number of associations between an miRNA and a disease's neighbors.
- *d*. *m*. *nb*: the number of associations between a disease and an miRNA's neighbors.
- *m*. *d*. *bt, m*. *d*. *cl*: betweenness, closeness of the node.

Feature matrix *X* = [*x*_1_*,…, x*_*i*_*,…, x*_*n*_]^T^ ϵ^ℝ^^n × *c*^ was generated by selecting both positive samples and negative samples with a ratio of 1:1 and putting them into a feature construction. The known associated miRNA-disease pairs were extracted from the HMDDv2.0 to compose the positive sample set, while the same number of unknown miRNA-disease pairs was randomly selected to constitute a negative sample set. The corresponding labels matrix *Y*=[*y*_1_*,…, y*_*j*_*,…, y*_*l*_]ϵℝ^n × *l*^, where the *j*-th category is 1 if *x*_*i*_ belongs to *j*-th category, and other categories are 0.

### Hypergraph Construction

A hypergraph is an extension of graph where an edge (i.e., a hyperedge) can connect more than two vertices and represent the structure of data via measuring the similarity between groups of different points. It has great advantages in complex data modeling. For any application using hypergraph learning approaches, the first step was to construct the corresponding hypergraph structure. Let *G* = (*V, E, W*) denote a hypergraph, which consists of a set of vertices *V* and a cluster of hyperedge *E* to which a corresponding weight matrix *W* is assigned. In this study, the total number of vertices was *n*, and each vertex represented an miRNA-disease pair in *X*. We used the K-nearest neighbor (KNN) algorithm and K-means algorithm to generate hyperedges, respectively. For KNN hypergraph *G*_1_, each time one vertex was selected as a centroid, and one hyperedge was constructed to connect the centroid with its *k* nearest neighbors in the corresponding feature space. For K-means hypergraph *G*_2_, we used the K-means algorithm to group all miRNA-disease pairs. If some miRNA-disease pairs are in the same group, they would have been linked by the corresponding edge.

A traditional hypergraph *G* could be denoted by a |*V*| × |*E*| incidence matrix *H*

(13) $$\begin{array}{l} h\left(v,\;e\right)\;=\{\begin{array}{c} 1\;if\;v\in e\\ 0\;if\;v\notin e \end{array} \end{array}$$

The degree of a vertex *v* ∈ *V* was obtained by

(14) $$\begin{array}{l} d\left(v\right)\;=\underset{e\in E}{\sum }w\left(e\right)h\left(v,e\right) \end{array}$$

and the degree of a hypergraph *e* ∈ *E* was obtained by

(15) $$\begin{array}{l} \delta \left(e\right)\;=\underset{\;v\in V}{\sum }h\left(v,e\right) \end{array}$$

*Dv* denoted the diagonal degree matrix of each vertex, and *De* denoted the diagonal matrix containing the degree of hyperedge.

For the hypergraph *G*_1_, the weight *w*_1_ of a hyperedge *e* was estimated by the sum of the distance between two vertexes in the same hyperedge

(16) $$\begin{array}{lll} w_{1}\left(e\right)\; & = & \;\underset{u\in e\;}{\sum }dist\left(v,u\right) \end{array}$$

(17) $$\begin{array}{lll} dist\left(v,\;u\right)\; & = & \exp\left(-∥x_{v}-\;x_{u}{∥}^{2}/\sigma^{2}\right) \end{array}$$

(18) $$\begin{array}{lll} \sigma \; & = & \sqrt{\frac{1}{n-1}\overset{n}{\underset{i=1}{\sum }}∥x_{i}-x_{bar}{∥}^{2}\;},\;x_{bar}=\;\frac{1}{n}\overset{n}{\underset{i=1}{\sum }}x_{i} \end{array}$$

where *v* was the centroid of *e* and *u* was *v*'s neighbor.

For the hypergraph *G*_2_, all the hyperedges were initialized with an equal weight, e.g., *w*_2_(*e*)=1/*n*_*e*_, where *n*_*e*_ was the number of hyperedges.

### Combinative Hypergraph Learning

**Figure 1:**
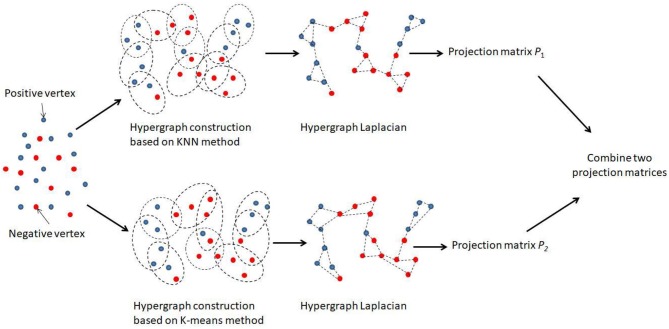
Flowchart of the combinative hypergraph learning to predict the association between miRNAs and diseases.

There were two hypergraphs in total, denoted by *G*_1_ = (*V*_1_, *E*_1_*, W*_1_) and *G*_2_ = (*V*_2_, *E*_2_, *W*_2_). For each hypergraph, we aimed to learn an individual projection matrix *P*_*i*_, and the overall combination of all projected matrices could be used to predict the disease-related miRNAs. Figure 1 illustrated the main framework of our method. We noted that an optimal combination of different hypergraph was also important. Thus, the combination weights *B* = [β_1_, β_2_] were further introduced as another objective of the learning task, where β_*i*_ was the combination weight for the *i*-th hypergraph subjecting to $\overset{2}{\underset{i=1}{\sum }}\beta_{i}=1$ and *B* ≥ 0.

We adopted the objective function proposed in Zhang et al. (2018):

(19) $$\begin{array}{r} \arg\;min_{Pi,\;B\;\geq \;0}\;\{\sum_{i=1}^{2}\beta_{i}\{\Omega \;(P_{i})+\;\lambda R_{emp}\;(P_{i})\;+\mu \Phi (P_{i})\;\}\\ \left\{+\eta \psi (B)\right\}\\ s.t.\;\sum_{i=1}^{2}\beta_{i}=1 \end{array}$$

Specifically, hypergraph Laplacian regularizer Ω(*P*_*i*_) was calculated as

(20) $$\begin{array}{l} \Omega \;(P_{i})\;=\frac{1}{2}\sum_{k=1}^{l}\sum_{e\in E_{i}}\sum_{u,v\in V_{i}}\frac{W_{i}\left(e\right)H_{i}\left(u,e\right)H_{i}\left(v,e\right)}{\delta_{i}\left(e\right)}(\frac{\left(XP_{i})(u,k\right)}{\sqrt{d_{i}(u)}}\\ \;-\frac{\left(XP_{i})(v,k\right)}{\sqrt{d_{i}(v)}}{)}^{2}\\ \;=\;tr(P_{i}^{T}X^{T}\Delta_{i}XP_{i}) \end{array}$$

where $\Delta_{i}=\;I-{D_{v}}_{i}^{-\left(1/2\right)}H_{i}W_{i}{D_{e}}_{i}^{-1}H_{i}^{T}{D_{v}}_{i}^{-\left(1/2\right)}\;$was the hypergraph laplacian matrix, in which *I* denoted the identity matrix, function *tr*(·) returned the trace of matrix.

The empirical loss term on *P*_*i*_ was denoted as

(21) $$\begin{array}{l} R_{emp}\;\left(P_{i}\right)\;=\;∥XP_{i}\;-Y{∥}^{2} \end{array}$$

Φ(*P*_*i*_) was a *l*_2_ norm regularizer to avoid over-fitting for *P*_*i*_. Φ(*P*_*i*_) was denoted as:

(22) $$\begin{array}{l} \Phi (P_{i})=\;∥P_{i}{∥}^{2} \end{array}$$

Here, Ψ(*B*) was measured as *l*_2_ norm of the hypergraph weights:

(23) $$\begin{array}{l} \text{Ψ}\left(B\right)\;=\;∥B{∥}^{2} \end{array}$$

The Equation (19) was a multiple variables optimization problem. We noted that it can be split into three independent sub-problems, which were related to each *P*_*i*_ and *B*, respectively. Therefore, to solve the optimization problem, we first optimized each *P*_*i*_ individually, and then optimized the combination weight *B*.

Firstly, we optimized each *P*_*i*_ individually. For each hypergraph, the learning task could be rewritten as

(24) $$\begin{array}{l} \arg\min_{P_{i}}\;\left\{\;tr\left(P_{i}^{T}X^{T}\Delta_{i}XP_{i}\right)\;+\;\lambda ∥\;XP_{i}\;-\;Y{∥}^{2}\;+\;\mu ∥\;P_{i}\;{∥}^{2}\;\right\} \end{array}$$

To solve the optimization task in Equation (24), we derived function to *P*_*i*_. The result could be mathematically denoted as follows

(25) $$\begin{array}{l} P_{i}\;=\;\lambda {\left(X^{T}\Delta_{i}X\;+\;\lambda X^{T}X\;+\;\mu I\right)}^{-1}X^{T}Y \end{array}$$

where *I* was an identity matrix.

Next, fix each *P*_*i*_ and optimized *B*. We let Θ_*i*_ = Ω(*P*_*i*_) + λ*R*_*emp*_ (*P*_*i*_) + μΦ(*P*_*i*_), and the learning task could be rewritten as

(26) $$\begin{array}{r} \arg\min_{B\;\geq \;0}\;\left\{\overset{2}{\underset{i=1}{\sum }}\beta_{i}\Theta_{i\;}+\eta ∥B{∥}^{2}\right\}\\ s.t.\;\overset{2}{\underset{i=1}{\sum }}\beta_{i}=1 \end{array}$$

To solve this task, the Lagrange multiplier method was employed and the optimization problem was defined as:

(27) $$\begin{array}{l} \arg\min_{B,\;\varepsilon }\;\left\{\overset{2}{\underset{i=1}{\sum }}\beta_{i}\Theta_{i}\;+\eta ∥B{∥}^{2}\right\}+\varepsilon \left(\overset{2}{\underset{i=1}{\sum }}\beta_{i}-1\right) \end{array}$$

It was derived that

(28) $$\begin{array}{l} \varepsilon =\frac{-\sum_{i=1}^{2}\Theta_{i}-2\eta }{2},\;\beta_{i}=\frac{1}{2}+\frac{\sum_{i=1}^{2}\Theta_{i}}{4\eta }-\frac{\Theta_{i}}{2\eta } \end{array}$$

According to the learned *P*_*i*_ and β_*i*_, the association score of the uncertain miRNA-disease pair *x*^un^ could be obtained by

(29) $$\begin{array}{l} \text{S}\left(x^{\text{un}}\right)\;=\overset{2}{\underset{i=1}{\sum }}\beta_{i}x^{\text{un}}.P_{i}\; \end{array}$$

## Results

### Cross Validation

**Figure 2:**
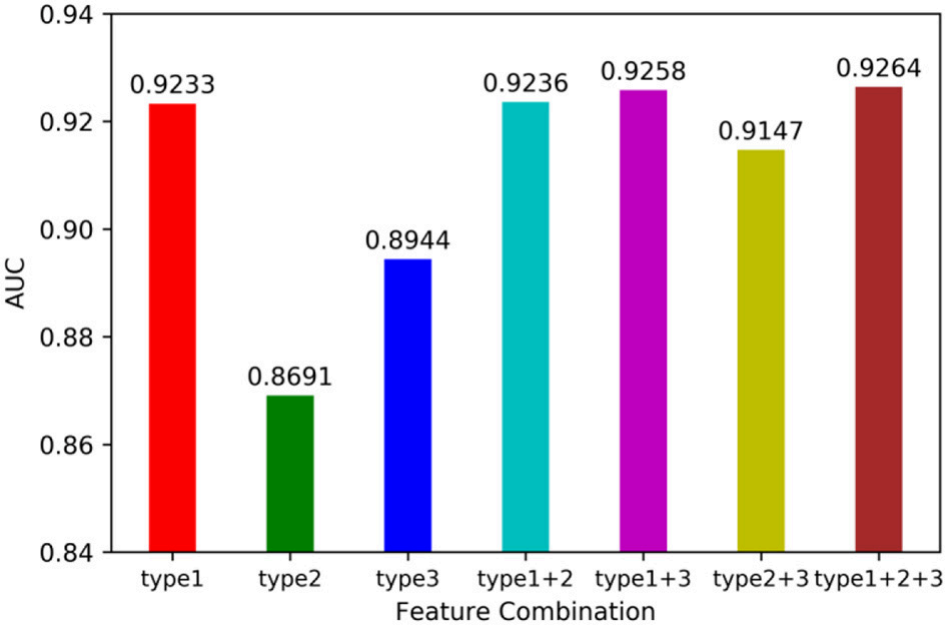
Influence of feature combination on model prediction accuracy.

We utilized leave-one-out cross validation (LOOCV) and 5-fold cross validation (5-CV) to evaluate the performance of MSCHLMDA. A typical machine learning task is to predict the label of a sample by its features. But for a particular learning algorithm, it is unknown which feature is effective. Therefore, it is necessary to select the relevant features that are beneficial to the learning algorithm from all the possible features. In this study, we combined three types of features arbitrarily, forming seven combinations, i.e., type 1; type 2; type 3; type 1, and type 2; type 1 and type 3; type 2 and type 3; type 1, type 2 and type 3. Then we conducted the 5-CV on each combination and calculated the area under curve (AUC) value. The results are shown in Figure 2. Our results indicate that when all three types of features were combined together, the AUC value was the highest. Therefore, for each miRNA-disease pair, we combined type 1, 2, and 3 features into one effective feature vector *x*, which was used to create the hypergraph to predict miRNA-disease associations.

**Figure 3:**
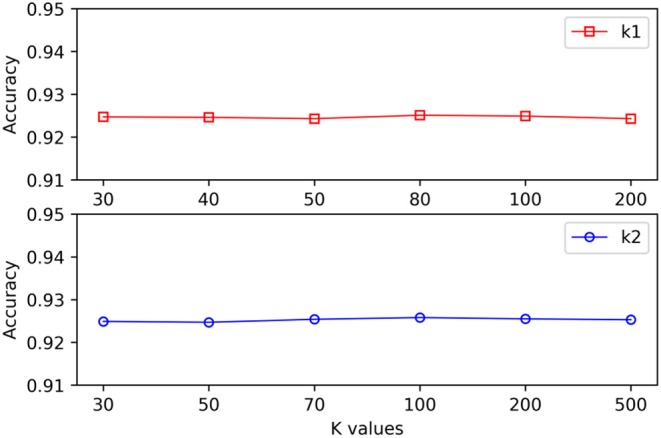
The effect of varying *k* values on the MSCHLMDA performance.

When different hypergraphs were created, *k*_1_ was adopted to represent the number of neighbors for each vertex, and *k*_2_ was adopted to represent the number of clusters. It is challenging to select the best *k* value, and thus different *k* values were used in this study to verify the impact of each value. As shown in Figure 3, it is observed that the proposed method could still obtain stable results even when *k*_1_ and *k*_2_ exhibited substantial changes.

**Figure 4:**
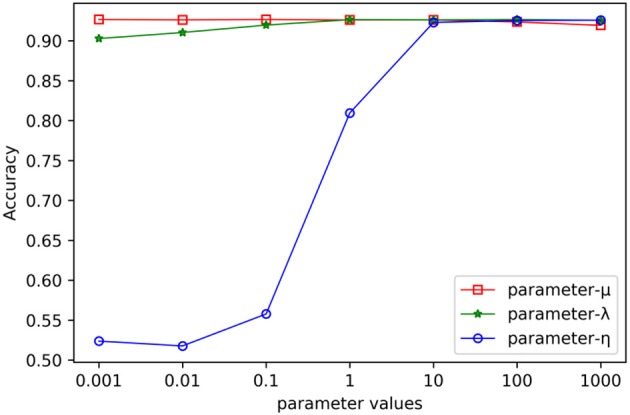
The effect of varying the parameters on the MSCHLMDA performance.

In the process of combinative hypergraph learning, the parameters λ, μ and η were the empirical loss, the regularizer on the projection matrices and the regularizer on the hypergraph weights, respectively. They were obtained from the set {10^−3^, 10^−2^, 10^−1^, 10^0^, 10^1^, 10^2^, 10^3^} by cross validating the values of various parameters. We first empirically set them as 10^0^, 10^0^, and 10^3^, respectively. When the influence of one parameters (such as λ) on the prediction performance was being verified, the other two parameters were fixed (such as μ =10^0^, η =10^3^) while the values of λ were changed from 10^−3^ to 10^3^. Figure 4 shows the AUC values with varying parameters under cross validation. Our results suggest that the proposed method could achieve relatively stable performance even if λ and μ show in a large range of variability, and that η had a greater impact on the results obtained from this method. It is found that MSCHLMDA achieved the best performance when η = 10^3^. Besides, we ensured more stable results by setting λ to 10^1^ and μ to 10^0^.

**Figure 5:**
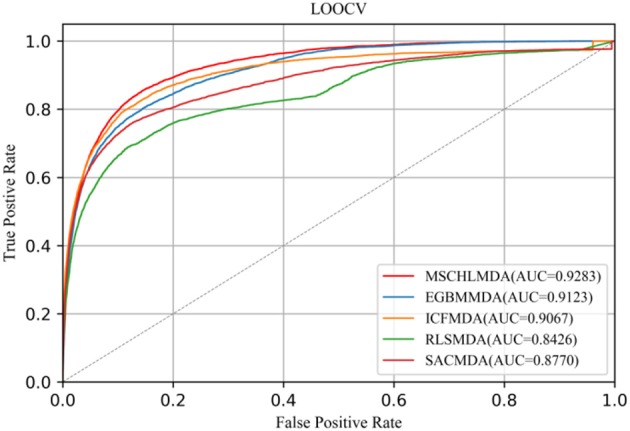
AUC of LOOCV compared with EGBMMDA,ICFMDA, RLSMDA, and SACMDA.

LOOCV considered each known association as a test sample, while remaining known associations were treated as the training set and all unknown associations were used as candidate samples. When MSCHLMDA completed the forecasting task, the scores of the test sample and candidate samples were compared to iteratively obtain a predicted ranking. The prediction was considered true positive if the rank of the test sample was no lower than the threshold. The prediction was considered false positive if the rank of the candidate sample was no lower than the threshold. The methods of EGBMMDA (Chen et al., 2018b), ICFMDA (Jiang et al., 2018), RLSMDA (Chen and Yan, 2014), and SACMDA (Shao et al., 2018) were implemented on the same dataset, and the parameters were set according to the values given in the original article. Finally, MSCHLMDA obtained the AUC of 0.9283 in LOOCV as shown in Figure 5 The AUCs of ICFMDA,EGBMMDA, SACMDA and RLSMDA in LOOCV are 0.9067, 0.9123, 0.8770, and 0.8426, respectively.

**Figure 6:**
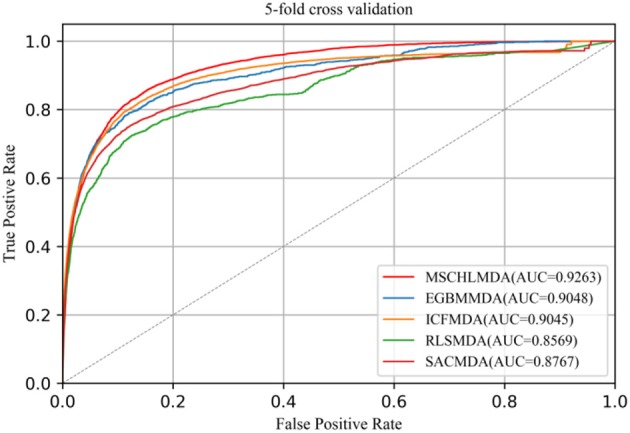
AUC of 5-fold cross validation compared with EGBMMDA, ICFMDA, SACMDA, and RLSMDA.

In 5-CV, all confirmed associations were randomly divided into five uncrossed subsets with equal sizes. One subset was considered as a test sample and the remaining four subsets as training sets. In this study, we implemented 5-CV 100 times to reduce the bias introduced by random divisions and then calculated the mean and standard deviation of AUCs. The average AUCs of MSCHLMDA, EGMMDA, ICFMDA, SACMDA, and RLSMDA are 0.9263 (+/−0.0006), 0.9048 (+/−0.0012), 0.9045 (+/−0.0008), 0.8767 (+/−0.0011), and 0.8569 (+/−0.0020), respectively (see Figure 6).

### Case Studies

To further evaluate the ability of MSCHLMDA to discover potential miRNA-disease associations, case studies of several important human diseases were carried out, such as prostate neoplasms, hepatocellular carcinoma and breast neoplasms. All confirmed associations in the HMDD v2.0 were put into the training set of MSCHLMDA. According to their prediction scores, the top 50 predicted miRNAs were selected, which were associated with the investigated disease. The other databases, namely dbDEMC (Yang et al., 2010) and miR2Disease (Jiang et al., 2009), were used to validate these findings.

The first experiment was implemented on prostate neoplasms. Prostate neoplasms, also known as the carcinoma of the prostate, are cancers developed from the prostate. The incidence of prostate cancer is 60% higher and the mortality rate is two to three times greater in black vs. white men (Sathekge et al., 2017). Early detection is substantially important for the treatment of prostate tumors. We used MSCHLMDA to predict miRNAs related to prostate neoplasms and considered them as candidate miRNAs. Then, all the candidate miRNAs were ranked in descending order by their predicted scores. Overall, 43 out of the top 50 miRNA predictions were verified by dbDEMC and miR2Disease (See Table 1).

**Table 1:** The top 50 predicted miRNAs associated with Prostate Neoplasms.

**miRNA**	**Evidence**	**miRNA**	**Evidence**
hsa-mir-21	miR2Disease;dbDEMC	hsa-mir-223	miR2Disease;dbDEMC
hsa-mir-155	dbDEMC	hsa-mir-133b	dbDEMC
hsa-mir-146a	miR2Disease	hsa-mir-146b	Unconfirmed
hsa-mir-221	miR2Disease;dbDEMC	hsa-mir-181a	miR2Disease;dbDEMC
hsa-mir-122	Unconfirmed	hsa-mir-124	dbDEMC
hsa-mir-16	miR2Disease;dbDEMC	hsa-mir-106b	dbDEMC
hsa-mir-29a	miR2Disease;dbDEMC	hsa-mir-203	Unconfirmed
hsa-mir-15a	miR2Disease;dbDEMC	hsa-let-7a	miR2Disease;dbDEMC
hsa-mir-1	dbDEMC	hsa-mir-196a	dbDEMC
hsa-mir-34a	miR2Disease;dbDEMC	hsa-mir-200b	Unconfirmed
hsa-mir-29b	miR2Disease;dbDEMC	hsa-mir-206	dbDEMC
hsa-mir-133a	dbDEMC	hsa-mir-19b	miR2Disease;dbDEMC
hsa-mir-143	miR2Disease;dbDEMC	hsa-mir-96	miR2Disease;dbDEMC
hsa-mir-126	miR2Disease;dbDEMC	hsa-mir-200c	dbDEMC
hsa-mir-222	miR2Disease;dbDEMC	hsa-mir-181b	miR2Disease;dbDEMC
hsa-mir-31	miR2Disease;dbDEMC	hsa-mir-214	miR2Disease;dbDEMC
hsa-mir-20a	miR2Disease	hsa-mir-34c	dbDEMC
hsa-mir-17	miR2Disease	hsa-mir-195	miR2Disease;dbDEMC
hsa-mir-142	Unconfirmed	hsa-mir-210	miR2Disease
hsa-mir-29c	dbDEMC	hsa-mir-24	miR2Disease;dbDEMC
hsa-mir-92a	Unconfirmed	hsa-mir-18a	Unconfirmed
hsa-mir-199a	miR2Disease;dbDEMC	hsa-let-7b	miR2Disease;dbDEMC
hsa-mir-150	dbDEMC	hsa-mir-148a	miR2Disease
hsa-mir-182	miR2Disease;dbDEMC	hsa-mir-19a	dbDEMC
hsa-mir-15b	dbDEMC	hsa-mir-200a	dbDEMC

In the second experiment using case studies, hepatocellular carcinoma was selected as an example to prove the ability of MSCHLMDA in predicting previously unreported miRNA-disease associations. Hepatocellular carcinoma is a primary liver cancer with a high mortality rate. It is one of the most common malignancies worldwide, especially in Asia, Africa, and southern Europe (Torre et al., 2015). In the first step, all the known hepatocellular carcinoma related miRNAs were removed. Only other disease similarity information and other disease-related miRNAs were used to reveal potentially related miRNAs for hepatocellular carcinoma. When the prediction task was complete, all the miRNAs based on their predicted association scores were prioritized. Finally, 49 out of the top 50 miRNAs were validated by HMDD v2.0, dbDEMC and miR2Disease (See Table 2).

**Table 2:** The top 50 predicted miRNAs associated with Hepatocellular Carcinoma.

**miRNA**	**Evidence**	**miRNA**	**Evidence**
hsa-mir-21	HMDD;miR2disease	hsa-mir-15b	HMDD;dbDEMC
hsa-mir-155	HMDD;miR2disease;dbDEMC	hsa-mir-92a	HMDD;miR2disease
hsa-mir-146a	HMDD;miR2disease;dbDEMC	hsa-mir-181a	HMDD;miR2disease;dbDEMC
hsa-mir-125b	HMDD;miR2disease	hsa-mir-182	HMDD;miR2disease
hsa-mir-122	HMDD;miR2disease;dbDEMC	hsa-mir-200b	HMDD;miR2disease
hsa-mir-221	HMDD;miR2disease;dbDEMC	hsa-mir-133b	HMDD
hsa-mir-29a	HMDD;dbDEMC	hsa-let-7a	HMDD;miR2disease;dbDEMC
hsa-mir-34a	HMDD;miR2disease;dbDEMC	hsa-mir-206	Unconfirmed
hsa-mir-16	HMDD;miR2disease;dbDEMC	hsa-mir-196a	HMDD
hsa-mir-1	HMDD;miR2disease	hsa-mir-200a	HMDD;miR2disease;dbDEMC
hsa-mir-15a	HMDD;miR2disease;dbDEMC	hsa-mir-124	HMDD;miR2disease
hsa-mir-133a	miR2disease	hsa-mir-146b	HMDD
hsa-mir-29b	HMDD;dbDEMC	hsa-mir-210	HMDD;dbDEMC
hsa-mir-145	HMDD;miR2disease;dbDEMC	hsa-mir-195	HMDD;miR2disease;dbDEMC
hsa-mir-199a	HMDD;miR2disease;dbDEMC	hsa-mir-214	HMDD;miR2disease;dbDEMC
hsa-mir-126	HMDD;miR2disease;dbDEMC	hsa-mir-34c	HMDD
hsa-mir-29c	HMDD;dbDEMC	hsa-mir-19b	HMDD;miR2disease
hsa-mir-20a	HMDD;miR2disease;dbDEMC	hsa-mir-18a	HMDD;miR2disease;dbDEMC
hsa-mir-150	HMDD;miR2disease;dbDEMC	hsa-mir-9	miR2disease
hsa-mir-17	HMDD;miR2disease	hsa-mir-19a	HMDD;miR2disease;dbDEMC
hsa-mir-31	HMDD;miR2disease	hsa-mir-106b	HMDD;miR2disease;dbDEMC
hsa-mir-222	HMDD;miR2disease;dbDEMC	hsa-mir-181b	HMDD;miR2disease;dbDEMC
hsa-mir-143	miR2disease;dbDEMC	hsa-let-7b	HMDD;miR2disease
hsa-mir-223	HMDD;miR2disease	hsa-mir-148a	HMDD;miR2disease;dbDEMC
hsa-mir-142	HMDD;miR2disease	hsa-mir-24	HMDD;miR2disease

In the final case study, our model was fitted with the miRNA-disease association dataset from HMDD v1.0, which is the old version of HMDD v2.0 and contains less information of miRNA-disease associations. This case study was used to demonstrate MSCHLMDA's robust prediction ability compared to various other datasets. Breast neoplasms were selected as our target disease. Breast neoplasms are the most common malignancies in women, it is also the second leading cause of cancer death among women after lung cancer (Desantis et al., 2016). Here, the whole prediction process was similar to the first experiment of case study. Eventually, 49 out of the top 50 miRNAs in our methods were verified by HMDD v2.0, dbDEMC and miR2Disease (See Table 3).

**Table 3:** The top 50 predicted miRNAs associated with Breast Neoplasms.

**miRNA**	**Evidence**	**miRNA**	**Evidence**
hsa-let-7i	HMDD;miR2Disease;dbDEMC	hsa-mir-32	dbDEMC
hsa-let-7e	HMDD;dbDEMC	hsa-mir-448	dbDEMC
hsa-mir-223	HMDD;dbDEMC	hsa-mir-29c	HMDD;miR2Disease;dbDEMC
hsa-let-7c	HMDD;dbDEMC	hsa-mir-181a	HMDD;miR2Disease;dbDEMC
hsa-mir-126	HMDD;miR2Disease;dbDEMC	hsa-mir-150	dbDEMC
hsa-let-7b	HMDD;dbDEMC	hsa-mir-30e	Unconfirmed
hsa-mir-182	HMDD;miR2Disease;dbDEMC	hsa-mir-30a	HMDD;miR2Disease
hsa-mir-191	HMDD;miR2Disease;dbDEMC	hsa-mir-98	miR2Disease;dbDEMC
hsa-mir-92b	dbDEMC	hsa-mir-203	HMDD;miR2Disease;dbDEMC
hsa-mir-101	HMDD;miR2Disease;dbDEMC	hsa-mir-199b	HMDD;dbDEMC
hsa-mir-130a	dbDEMC	hsa-mir-659	dbDEMC
hsa-mir-532	dbDEMC	hsa-mir-521	dbDEMC
hsa-mir-16	HMDD;dbDEMC	hsa-mir-23b	HMDD;dbDEMC
hsa-let-7g	HMDD;dbDEMC	hsa-mir-130b	dbDEMC
hsa-mir-373	HMDD;miR2Disease;dbDEMC	hsa-mir-196b	dbDEMC
hsa-mir-92a	HMDD	hsa-mir-335	HMDD;miR2Disease;dbDEMC
hsa-mir-24	HMDD;dbDEMC	hsa-mir-26a	HMDD;miR2Disease;dbDEMC
hsa-mir-99b	dbDEMC	hsa-mir-224	HMDD;dbDEMC
hsa-mir-18b	HMDD;dbDEMC	hsa-mir-192	dbDEMC
hsa-mir-15b	dbDEMC	hsa-mir-195	HMDD;miR2Disease;dbDEMC
hsa-mir-99a	dbDEMC	hsa-mir-328	HMDD;miR2Disease;dbDEMC
hsa-mir-372	dbDEMC	hsa-mir-135a	HMDD;dbDEMC
hsa-mir-106a	dbDEMC	hsa-mir-27a	HMDD;miR2Disease;dbDEMC
hsa-mir-520b	HMDD;dbDEMC	hsa-mir-452	HMDD;dbDEMC
hsa-mir-100	HMDD;dbDEMC	hsa-mir-186	dbDEMC

In conclusion, our results show the reliable prediction ability of MSCHLMDA, indicating that MSCHLMDA could be a useful computational mode to investigate a potential disease-related miRNAs association.

## Discussion

In recent years, finding novel miRNAs associated with specific diseases has attracted increasing attention in understanding the pathophysiology of the diseases and discovery of new drugs to establish effective treatment strategies. In this study, we proposed a combinative hypergraph learning (CHL) method called MSCHLMDA to effectively define miRNA/disease similarity for predicting underlying miRNA-disease associations. CHL captures the similarity between two samples in the same category by KNN hypergraph and K-means hypergraph. MSCHLMDA's performance was verified by cross validation and case studies. These results indicate that MSCHLMDA is able to generate reliable candidate miRNA-disease associations for further validation by biologists.

The improved performance of our model could be mainly attributed to the following two aspects. First, an informative feature vector was created from a statistical analysis and a graph theoretic. The statistical features recorded the sum, the mean, the histogram distributions of the similarity scores, the neighbor count and the neighbor's similarity scores. For miRNAs and diseases, the graph theoretic features contained the betweenness and closeness centrality measures of the network graphs. Second, we used hypergraph learning to design a predictive model. Hypergraph-based models have proven to be beneficial for a variety of classification/clustering tasks, because it can represent the information that three or more vertices have the same semantic attribute, which common graphs are unable to describe. Hypergraphs can model the high-order relationships between their vertices by hyperedges, whose influence can be assessed by properly estimating their weights. Furthermore, we employed the neighborhood-based formulation and the clustering techniques to generate the hyperedges.

In our previous model of HGMDA (Wu et al., 2019), we also used hypergraph learning, but there are many differences between the implementation process of these two models. First, the hypergraph construction was different. In HGMDA, we only used the K-means algorithm for clustering, which means that known miRNA-disease associations were not utilized to extract the clustering relationship of miRNA-disease pairs. In the current study, KNN and a K-means algorithm was used to seek the relationship between miRNA-disease pairs, which was more comprehensive because KNN was a supervised learning method. Second, the weights of the hyperedges were different. To generate a better hypergraph representation, different hyperedges should have different influences. In HGMDA, all hyperedges had the same weight failing to reflect the importance of different hyperedges. However, in this work, we assigned different weights to each hyperedge based on the distance of each vertex from its neighborhood; this can help to improve the representation ability of the hypergraph structure. Third, the projection matrix was different. In HGMDA, it was required to iterate multiple times to get a stable projection matrix, while in this work we could obtain two projection matrices directly, then combine them into a comprehensive mapping matrix, which was scored higher in efficiency and accuracy.

This method still has some limitations. First, it is required to add negative samples in the training datasets to train the predictive model. Second, due to the computational cost of the hypergraph construction, our method fails to efficiently deal with large-scale samples. Besides, with newly discovered miRNAs, the originally learned projection matrices may be unable to represent the data distribution well. These shortages limit the application range of our model. In future study, we will further investigate the online updates of the learned hypergraph embedding results.

## Data Availability Statement

All datasets generated for this study are included in the article/supplementary material.

## Author Contributions

JN and CZ conceived and supervised the entire project. QW developed the prediction method. YW and ZG undertook data collection and designed the experiments. QW, YW, and ZG analyzed the result. JN, CZ, and QW wrote the paper. All authors read and approved the final manuscript.

### Conflict of Interest

The authors declare that the research was conducted in the absence of any commercial or financial relationships that could be construed as a potential conflict of interest.
